# The Gene Expression of the Transcription Factors HY5 and HFR1 Is Involved in the Response of *Arabidopsis thaliana* to Artificial Sun-like Lighting Systems

**DOI:** 10.3390/biology14101315

**Published:** 2025-09-23

**Authors:** Peter Beatrice, Gustavo Agosto, Alessio Miali, Donato Chiatante, Antonio Montagnoli

**Affiliations:** Department of Biotechnology and Life Sciences, University of Insubria, Via Monte Generoso, 71, Padiglione Spallanzani, 21100 Varese, Italydonato.chiatante@uninsubria.it (D.C.); antonio.montagnoli@uninsubria.it (A.M.)

**Keywords:** CoeLux^®^, LED-sourced, photomorphogenesis, biophilia, light intensity, light spectrum

## Abstract

Plants can sense light signals using specific photoreceptors, activating light signaling pathways to precisely regulate photomorphogenesis and shade-avoidance responses. This study examines the molecular responses of *Arabidopsis thaliana* to the CoeLux^®^ lighting system, a unique LED-based light source designed to simulate natural sunlight. Previous studies found that the CoeLux^®^ light type, characterized by a higher blue-to-green ratio and reduced blue light levels, stimulates responses in plants comparable to those displayed in shade conditions. This research compared the effects of CoeLux^®^ lighting to conventional high-pressure sodium (HPS) lamps, focusing on the expression of critical photomorphogenesis-related genes under both long- and short-term light treatments. Lower HY5 and elevated HFR1 expression levels were observed under the CoeLux^®^ light type and low-intensity light conditions. On the contrary, the influence of the CoeLux^®^ light type on COP1 and PIFs expression levels seems more marginal. These responses suggest a complex regulation involving both gene expression and protein-level adjustments. Additionally, mutant plants lacking these essential regulatory genes displayed altered morphologies under CoeLux^®^ light, underscoring the functional contribution of these genes in the adaptation to light. Our findings are twofold, advancing the understanding of plant–light relationships and plant adaptation to artificial light environments. These may foster strategies for optimizing indoor plant growth under simulated sunlight conditions.

## Simple Summary

Plants rely on light not only for photosynthesis but also to regulate their growth and development. This study investigated how *Arabidopsis thaliana*, a model plant species, responds at the molecular level to CoeLux^®^, an innovative artificial lighting system that closely mimics natural sunlight. Unlike conventional lights used in plant growth, CoeLux^®^ produces a unique light spectrum with reduced blue light and a higher blue-to-green ratio, creating conditions that resemble light found in shaded environments. Our findings show that CoeLux^®^ lighting influences the activity of key genes involved in light perception and plant development, leading to changes similar to those seen in low-light or shaded conditions. Plants lacking some of these genes showed altered growth under CoeLux^®^ light, confirming their role in adapting to this artificial environment. This research helps deepen our understanding of how plants react to artificial light sources and can support better design of indoor growing systems for both plant health and human well-being.

## 1. Introduction

Plants are photoautotrophic and immobile organisms that rely on light for survival [1]. To cope with changing light conditions and to properly balance growth and energy use, they continuously monitor the quantity, quality, and direction of incoming light [2]. This is achieved through several photoreceptor proteins that detect a wide range of light signals, including UV-B to far-red light. These light signals regulate numerous cellular and physiological processes, enhancing photosynthesis, reducing photodamage, and influencing the plant’s growth and structure [3]. In higher plants, three main families of photoreceptors have been recognized: phytochromes, cryptochromes, and phototropins [1]. Phytochromes (PHYs) function mainly as red and far-red light sensors and are key regulators of multiple developmental processes in plants, such as germination, de-etiolation, root and stomatal development, flowering, and shade-avoidance responses [3]. Cryptochromes (CRYs), acting as UV-A/blue light photoreceptors, participate in several growth and physiological pathways, including hypocotyl and stem elongation, de-etiolation, leaf and root growth, anthocyanin biosynthesis, circadian rhythm control, and the regulation of flowering [4]. Phototropins (PHOTs), also UV-A and blue light receptors, control phototropism in shoots and roots and regulate leaf shape, stomatal opening, chloroplast accumulation, and lateral root elongation [5]. Additionally, UV-B signals are detected by the UV resistance locus 8 protein (UVR8), which inhibits shade avoidance, hypocotyl elongation, petiole elongation, and rosette expansion [6].

The perception of light by photoreceptors activates many downstream transcription factors and signaling proteins that play crucial roles in regulating gene expression in plants. Among the proteins that have been functionally characterized in *Arabidopsis thaliana*, elongated hypocotyl 5 (HY5), long hypocotyl in far-red 1 (HFR1), constitutive photomorphogenesis 1 (COP1), phytochrome interacting factor 4 (PIF4), and phytochrome interacting factor 5 (PIF5) emerge as key players in the light signaling pathways. HY5 is the central hub of the transcriptional network that regulates photomorphogenesis downstream of phytochromes, cryptochromes, and UV-B photoreceptors. Unlike HY2 (involved in phytochrome chromophore biosynthesis [7]) and HY3/PHYB (a red-light photoreceptor [8]), HY5 is a bZIP transcription factor that is considered a master regulator in *A. thaliana*. It binds to the promoters of nearly 4000 light-inducible genes, thereby regulating a wide diversity of photomorphogenic responses [9], including key developmental processes such as cell elongation, cell proliferation, chloroplast development, pigment accumulation, and nutrient assimilation [10]. In addition to photoreceptors, a large number of proteins regulate HY5 function; among these, COP1 acts as a potent inhibitor of HY5 activity, alongside other signaling components, such as PIF4 and PIF5 [10]. COP1 is an E3 ubiquitin ligase that represses photomorphogenesis in the dark by targeting HY5 and other positive regulators for degradation [11]. HFR1 is also a transcription factor that acts as a positive regulator of photomorphogenesis and is ubiquitinated by COP1, thus marked for post-translational degradation [12]. PIF4 and PIF5 are bHLH transcription factors that optimize plant growth and development by integrating various environmental signals, including light and temperature, with hormonal signals, both transcriptionally and post-translationally [13,14,15]. PIF4 and PIF5 inhibit photomorphogenic development in darkness and regulate shade-avoidance responses, leaf senescence, and diurnal growth patterns. In the dark, COP1 acts synergistically with PIFs to destabilize HY5 and HFR1, thereby preventing photomorphogenesis. In response to red light exposure, COP1 promotes the ubiquitination and degradation of PIFs [15]. The interplay between these factors, with COP1’s regulatory role in degradation and the balance between HY5 and HFR1 activation and PIF4/PIF5 repression, orchestrates the plant’s growth and development in response to changing light conditions. The current literature describes the regulatory mechanisms involved in plants’ responses to weak light or light quality at the protein level. Although the main regulation of these genes occurs at the protein level, exploring the regulatory mechanisms at the transcriptional level could also contribute to advancing the understanding of plant–light relationships. The manipulation of light through artificial light systems may be a powerful tool in fostering our understanding of how plants respond to light and, specifically, plant responses to artificially lighted environments, an understanding which is crucial for implementing biophilic approaches in indoor settings. In particular, the biophilia hypothesis suggests that a lack of human connection with nature can significantly diminish health, well-being, and performance. Numerous studies have demonstrated that incorporating plants into office spaces can significantly enhance occupants’ attention, creativity, and productivity, while also reducing anxiety and nervousness [16,17]. Additionally, window views have been found to amplify these positive effects [18]. In this context, incorporating indoor plants alongside artificial lighting systems could provide a novel approach to enhancing the quality of life in enclosed environments that lack natural light. CoeLux^®^ is an advanced LED lighting technology designed to reproduce the visual impression of sunlight entering from above, giving the perception of a distant sun framed by a blue sky [19,20]. Previous studies have reported that this artificial skylight can elicit long-term psycho-physiological benefits in humans, comparable to those of natural daylight [21]. However, there is currently limited knowledge of how plants grow and adapt to this type of light, ensuring the maintenance of desired growth characteristics (e.g., healthy leaves, flowering ability, and uniform and compact foliage).

In our previous work, we analyzed the intensity and spectral characteristics of CoeLux^®^ lighting [22] and investigated its effects on the morphology and physiology of the model plant *A. thaliana* [22,23], as well as the aromatic species *Mentha* × *piperita* and *Ocimum basilicum* [24]. Relative to high-pressure sodium (HPS) lamps, CoeLux^®^ light exhibits a higher blue-to-green (B/G) ratio and lower overall blue light emission. Along with the low light intensity generated by the CoeLux^®^ lighting systems, these factors create conditions that partially resemble a shaded environment. Consequently, the phenotype of *A. thaliana* was marked by low biomass production, a small leaf area, and a low lamina-to-petiole-length ratio. At the same time, aromatic plants exhibited lower biomass production and larger leaf areas compared to control plants grown under high-pressure sodium (HPS) lighting. These data suggest the onset of responses similar to those characterizing shade avoidance, likely triggered by the spectral composition and the low intensity of the CoeLux^®^ light type. Furthermore, we analyzed the expression levels of the main photoreceptor genes of *A. thaliana*, suggesting the involvement of both PHYs, CRYs, and PHOTs, as well as the need for further research on downstream regulatory factors [25]. Analyzing the latter’s expression, this work aims to bridge the gap between the expression of photoreceptor genes and the morphological and physiological responses observed in *A. thaliana*. We hope that increasing knowledge about the molecular mechanisms underlying plant responses to CoeLux^®^ light will advance our understanding of the plant–light relationship and plant adaptation to artificial light environments, thereby fostering strategies for optimizing indoor plant growth under simulated sunlight conditions.

Based on the previous findings and the known photomorphogenesis-promoting functions of the *HY5* gene, we expect to observe (i) a lower expression level of this gene in *A. thaliana* plants grown under the CoeLux^®^ light type and in plants subjected to treatments characterized by lower light intensity. On the contrary, we expect to observe (ii) a higher expression level of the genes that suppress photomorphogenesis, i.e., *COP1*, *PIF4*, and *PIF5*, both in *A. thaliana* plants grown under the CoeLux^®^ light type and in plants growing under treatments characterized by lower light intensity. Furthermore, given the involvement of the *HFR1* gene in the suppression of shade-avoidance responses during long periods of suboptimal light conditions, we expect to observe (iii) a higher expression level of this gene both at low light intensities and in *A. thaliana* plants grown under the CoeLux^®^ light type. Finally, (iv) we expect that loss-of-function mutant plants of these critical regulatory genes will show a diverse phenotypic response under the two light types under study, highlighting potential functional contributions to plant development and in the response mechanisms to the CoeLux^®^ light type.

To test these hypotheses, *A. thaliana* plants were exposed to long- and short-duration light treatments under the CoeLux^®^ light type, and the gene expression levels of the selected transcription factors were evaluated at various light intensities and multiple time points following the exposure to the CoeLux^®^ light type.

## 2. Materials and Methods

### 2.1. Plant Material and Growth Conditions

Seeds of *Arabidopsis thaliana* Col-8 wild type (N60000) and the homozygous loss-of-function mutant line *hy5* (N679707) were acquired from the Eurasian *Arabidopsis* Stock Centre (NASC) [26]. Loss-of-function mutant lines *hfr1-4*, *pif4-101*, and *pif5* were generously provided by the University of Freiburg, from its seed collection. The seeds underwent a stratification process at 4 °C for 5 days on 1% agar gel before being transferred to pot cavities with a 5 cm diameter (Araflats; Arasystem; Ghent/Belgium) filled with sterilized soil-less substrate. The plants were cultivated at a temperature close to 22 °C and a photoperiod of 14 h.

### 2.2. Light Characteristics

**Figure 1:**
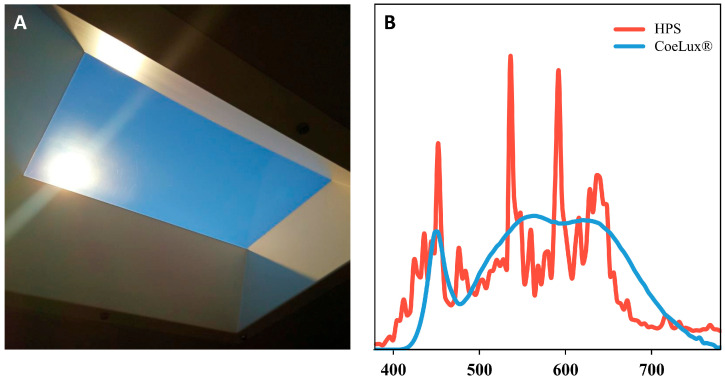
(**A**) The visual appearance of the 45HC CoeLux^®^ lighting system and (**B**) the spectral curves measured within the CoeLux^®^ (blue) and HPS (red) light types between 380 nm and 780 nm.

Two distinct light sources were employed: high-pressure sodium (HPS) lamps, commonly used in indoor plant production [27], and LED-sourced 45HC CoeLux^®^ systems designed to mimic natural sunlight (Figure 1A). These light sources were previously characterized, measuring both light intensity and spectra (Figure 1B) [22]. The HPS light type exhibits a complex spectrum characterized by multiple peaks, with four prominent peaks at 453, 536, 592, and 638 nm, resulting in higher blue and lower yellow and red components with respect to the CoeLux^®^ light type (Table 1). On the contrary, the CoeLux^®^ light type exhibits a sharp peak in the blue region (452 nm) and a broad peak between 490 and 700 nm, resulting in lower blue and higher yellow and red components with respect to the HPS light type (Table 1). Despite comparable values of far-red (FR) light, the red-to-far-red ratio (R/FR) is lower under the HPS light type (2.43) compared to the CoeLux^®^ light type (4.68), whereas the blue-to-green ratio (B/G) is higher under the HPS light type (0.83) than under the CoeLux^®^ light type (0.50) [22].

### 2.3. Light Treatments and Experimental Design

**Figure 2:**
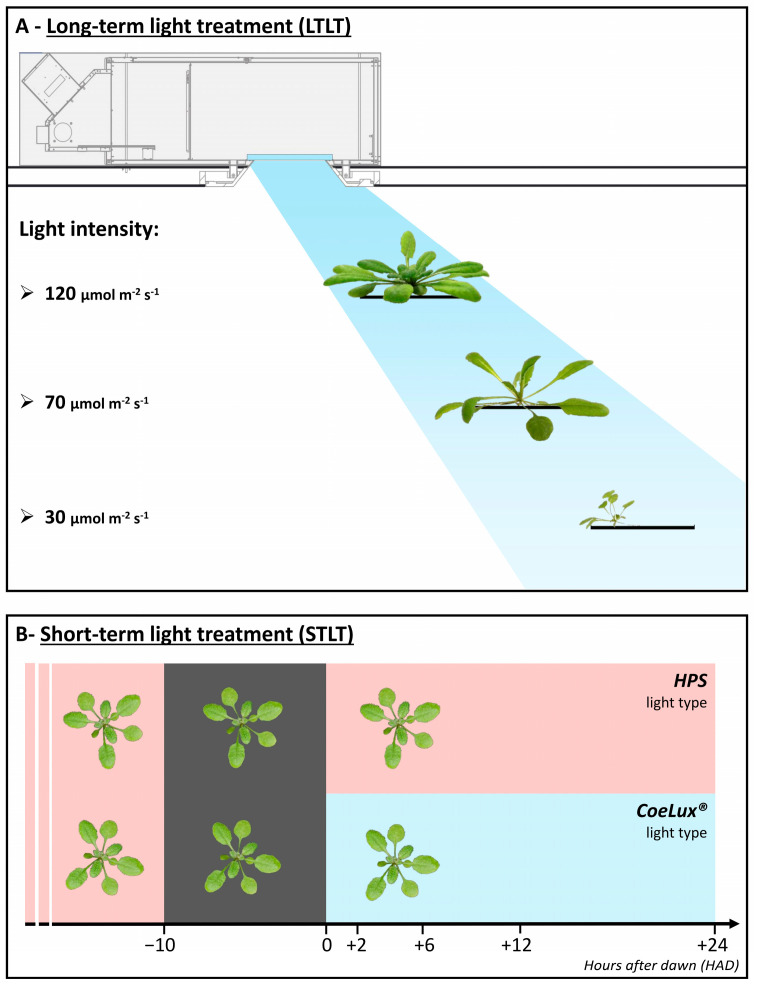
Schemes illustrating the experimental setup of the (**A**) long-term light treatment and the (**B**) short-term light treatment.

We exposed wild-type (WT) *A. thaliana* plants to two different light treatments, as reported in Ref. [25]. In the long-term light treatment (LTLT), plants were grown under the CoeLux^®^ light type at increasing distances from the light source (20, 85, and 205 cm), resulting in decreasing light intensities (120, 70, and 30 μmol m^−2^s^−1^; Figure 2A). Control plants were grown under the HPS light type at equal light intensities. To analyze the gene expression in plants at the same phenological stage, the fifth and sixth rosette leaves were sampled when they reached full expansion, which occurred between 17 and 30 days after sowing (DAS), depending on the light intensity. Leaves from six different plants were sampled 3 h after dawn (i.e., the light switch on; HAD), frozen in liquid nitrogen, and finely ground in a mortar to obtain a single biological replicate. Two pooled samples (biological replicates) were collected and analyzed independently. In the short-term light treatment (STLT), plants were grown until the six-leaf stage (17 DAS) under HPS light at 120 μmol m^−2^s^−1^. Samples for Time 0 were collected pre-dawn, as described above. Then, half of the plants were transferred under the CoeLux^®^ system for the light treatment at 120 μmol m^−2^s^−1^, while the other half remained under HPS lamps as a control (Figure 2B). Leaves number five and six after cotyledon expansion were sampled under both light sources at 2, 6, 12, and 24 HAD. Leaves from six different plants were pooled to form a single biological replicate, for a total of three biological replicates.

Finally, loss-of-function mutant plants (*hfr1*, *hy5*, *pif4*, and *pif5*) and their respective WT controls were grown for 25 days under both light types, at a light intensity of 120 μmol m^−2^s^−1^.

### 2.4. Morphological Leaf Traits Analysis

Mutant plants were sampled 25 days after sowing. Leaves and flower stalks were scanned (Epson Expression 12000XL; Seiko Epson Corporation; Suwa/Japan) at 800 dpi and dried at 70 °C until constant weight. The obtained images were analyzed with WinRhizo (Regent Instrument; Quebec City/QC/Canada) to estimate the projected rosette area (PRA; including both leaves and flower stalk) and with ImageJ v1.53u (National Institute of Health/NIH, Bethesda/MD/USA) to measure the lamina (L) and petiole length (P) of the two completely expanded younger leaves. The lamina-to-petiole-length ratio (L/P) was then calculated.

### 2.5. Gene Expression Levels Analysis

Total RNA was extracted using the RNeasy Plant Mini Kit (Qiagen, Venlo, The Netherlands), following the manufacturer’s instructions. RNA integrity was assessed via electrophoresis on a 1% agarose gel, and RNA concentration was measured spectrophotometrically with the NanoDrop 2000 (Thermo Scientific, Waltham, MA, USA). Contaminant genomic DNA elimination and cDNA synthesis were performed with the QuantiTect Reverse Transcription Kit (Qiagen). Primers with an exon–exon junction span were designed using the NCBI Primer-BLAST tool (Table 2). Real-time PCR reactions were conducted in a 20 μL reaction mix containing 10 μL 2× SensiFAST SYBR Mix (Meridian Bioscience, Cincinnati, OH, USA), 4.6 μL H_2_O, 0.4 μL primer mix (5 μM), and 5 μL cDNA (2 ng/μL). Amplification was performed with the CFX Maestro thermocycler (Bio-Rad, Hercules, CA, USA), configuring the following parameters: 95 °C for 10 min, 40 cycles of 95 °C for 15 s, and 60 °C for 60 s; and then 95 °C for 5 s, followed by melting analysis. Each reaction was performed in triplicate. Gene expression levels were normalized using the 2^−ΔΔCT^ method, with the geometric mean of three housekeeping genes (PP2AA3, UBQ10, and SAND) employed according to Ref. [25]. The low variability observed (CV < 3%) and shown in Supplementary Tables S1 and S2 confirms their suitability as housekeeping genes for normalizing the gene expression data in this study. For the relative gene expression analysis, in the LTLT, all treatments were normalized to the expression levels measured in *A. thaliana* plants grown under the HPS light type at 120 μmol m^−2^s^−1^. These plants were chosen as a reference, as this intensity and these spectra are considered the optimal conditions for *A. thaliana* growth, thus providing a biologically meaningful baseline for comparing deviations from optimal growth. In the STLT, we normalized all time points to the expression levels measured in plants sampled at pre-dawn (time 0), as this represents the starting condition and meaningful baseline for the subsequent time points.

### 2.6. Statistical Analysis

Statistically significant differences among the observed means (*p* < 0.05) were calculated with SPSS Statistics 25 (IBM) via the post hoc Dunnett’s T3 test for multiple comparisons. Only two biological replicates were used for the LTLT, which may reduce the statistical power of our analysis and increase the potential influence of biological variability on the results; however, the consistent trends observed suggest that the results remain informative. Differences within the HPS light type were denoted with the letters a, b, c, d, and e, while differences within the CoeLux^®^ light type were denoted with the letters z, y, x, and w. Differences between each CoeLux^®^ treatment and its respective HPS control were marked with an asterisk. Error bars represent the ±95% confidence interval (CI).

## 3. Results

### 3.1. Long-Term Light Treatment

**Figure 3:**
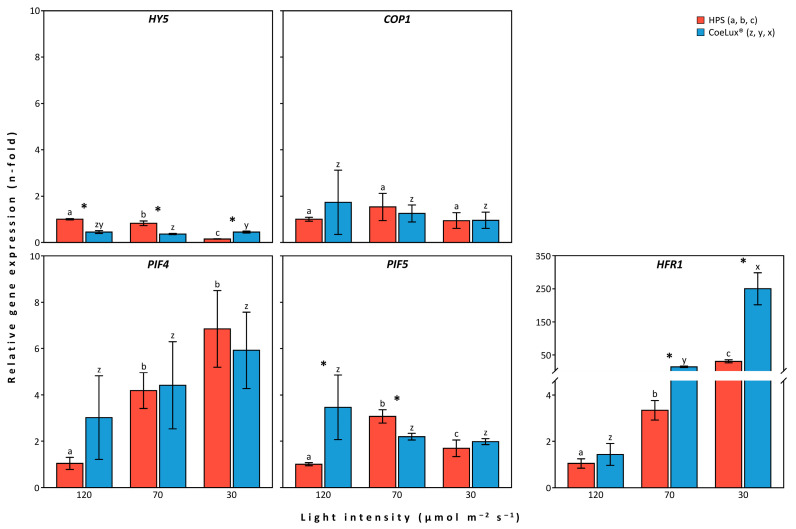
Relative gene expression levels measured in the LTLT. The gene expression levels of the presented genes are relative to those measured in *A. thaliana* plants grown under HPS light at 120 μmol m^−2^s^−1^. Data represent the means of *n* = 2 biological replicates ± 95% CI. Black asterisks represent statistically significant differences (*p* < 0.05) between plants grown under the two light types.

The *HY5* gene showed a different expression level pattern in plants grown under the two light types analyzed (Figure 3—*HY5*). In particular, under the HPS light type, the expression level of *HY5* decreased with a decrease in light intensity, reaching its lowest expression (0.1-fold) at 30 μmol m^−2^s^−1^. Under the CoeLux^®^ light type, the expression of the *HY5* gene was constant at all light intensities analyzed, ranging between 0.36- and 0.44-fold the reference plants (Supplementary Table S3). Regarding the HPS light type, significantly lower values were observed under the CoeLux^®^ light type at 120 and 70 μmol m^−2^s^−1^, while higher values were observed at 30 μmol m^−2^s^−1^.

The *COP1* gene showed minimal and non-significantly different expression level changes in response to the diverse light intensities and light types analyzed (Figure 3—*COP1*).

The *PIF4* gene showed a different expression level pattern in plants grown under the two light types analyzed (Figure 3—*PIF4*). In particular, under the HPS light type, the expression level of the *PIF4* gene significantly increased with a decrease in light intensity, reaching the highest expression (6.8-fold) at 30 μmol m^−2^s^−1^. Under the CoeLux^®^ light type, the expression level of the *PIF4* gene showed a similar pattern, with values ranging between 3.0- and 5.9-fold from the reference plants (Supplementary Table S3). However, due to the high variability of the data, no statistically significant differences were observed. Also, no significant differences were observed between the two light types analyzed.

The *PIF5* gene showed a different expression level pattern in plants grown under the two light types analyzed (Figure 3—*PIF5*). In particular, under the HPS light type, the expression level of the *PIF5* gene reached its highest and lowest values, respectively, at 70 μmol m^−2^s^−1^ (i.e., 3.0-fold) and 120 μmol m^−2^s^−1^, while an intermediate value was measured at 30 μmol m^−2^s^−1^ (i.e., 1.7-fold). Under the CoeLux^®^ light type, the expression level of the *PIF5* gene was constant at all light intensities analyzed, ranging between 2.0- and 3.5-fold that of reference plants. Statistically significantly higher and lower values were observed under the CoeLux^®^ light type, with respect to the HPS light type, respectively, at 120 and 70 μmol m^−2^s^−1^.

The *HFR1* gene showed a similar expression level pattern in plants grown under both light types analyzed (Figure 3—*HFR1*). Under both light types, the expression level of the *HFR1* gene strongly increased with the decrease in the light intensity, reaching a 31-fold expression at 30 μmol m^−2^s^−1^ under the HPS light type and a 250-fold expression under the CoeLux^®^ light type (Supplementary Table S3). Significantly higher values were observed under the CoeLux^®^ light type, compared to the HPS light type, at 70 and 30 μmol m^−2^s^−1^.

### 3.2. Short-Term Light Treatment

**Figure 4:**
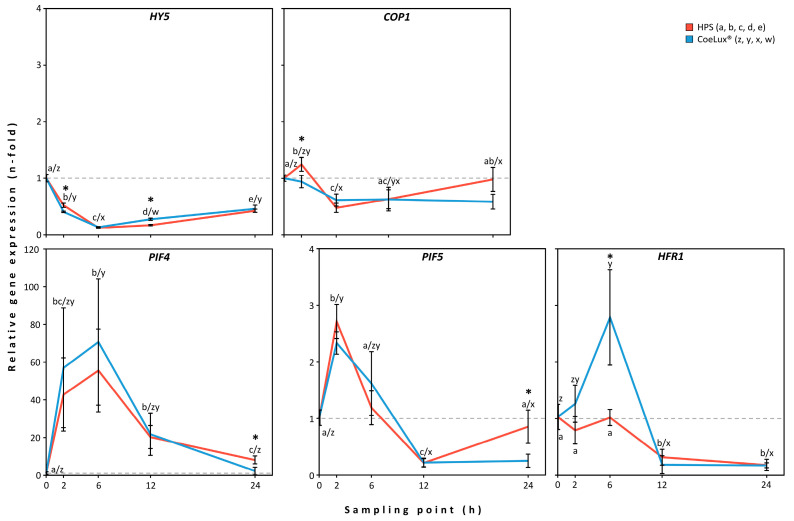
Relative gene expression levels measured in the STLT. The gene expression level measured in *A. thaliana* plants after 2, 6, 12, and 24 h of light treatment is relative to the expression level measured at the 0 h sampling point, which is set to 1 (dotted line) and was measured pre-dawn. The presented data represent the means of *n* = 3 biological replicates ± 95% CI. Black asterisks represent statistically significant differences (*p* < 0.05) between plants grown under the two light types.

The *HY5* gene showed a similar expression level pattern in plants grown under the two light types analyzed (Figure 4—*HY5*). The gene expression level rapidly decreased to 0.1-fold in the first 6 h after dawn (HAD) and slowly increased to 0.4-fold between 6 and 24 HAD (Supplementary Table S4). Statistically significant lower and higher values were observed under the CoeLux^®^ light type, with respect to the HPS light type, respectively, at 2 and 12 HAD.

The *COP1* gene showed a different expression level pattern in plants grown under the two light types analyzed (Figure 4—*COP1*). Under the HPS light type, the *COP1* gene showed an initial over-expression at 2 HAD, up to 1.2-fold the reference value, a subsequent reduction to 0.5-fold at 6 HAD, followed by an increase in the expression level that reached nearly the reference pre-dawn values (Supplementary Table S4). Under the CoeLux^®^ light type, the COP1 gene expression level slowly decreased, reaching 0.6-fold the reference value at 6 HAD, and then remained stable until 24 HAD. A statistically significantly lower value was observed under the CoeLux^®^ light type, with respect to the HPS light type, at 2 HAD.

The *PIF4* gene showed a similar expression level pattern in plants grown under the two light types analyzed (Figure 4—*PIF4*). The gene expression level increased significantly until 6 HAD, reaching 55.6-fold the reference value under the HPS light type and 70.7-fold under the CoeLux^®^ light type (Supplementary Table S4). Following this, the gene expression level decreased, reaching 8.1- (HPS) and 2.3-fold (CoeLux^®^) the reference value at 24 HAD. A statistically significantly lower value was observed under the CoeLux^®^ light type, with respect to the HPS light type, at 24 HAD.

The *PIF5* gene showed a similar expression level pattern in plants grown under the two light types analyzed (Figure 4—*PIF5*). In particular, the *PIF5* gene showed an initial over-expression at 2 HAD, up to 2.7- and 2.3-fold the reference value, and a subsequent reduction up to 0.2-fold at 12 HAD (Supplementary Table S4). Following, under the HPS light type, the gene expression level returned nearly to the pre-dawn values, while under the CoeLux^®^ light type, it remained stable at 0.2-fold the reference value. A statistically significantly lower value was observed under the CoeLux^®^ light type, with respect to the HPS light type, at 24 HAD.

The *HFR1* gene showed a different expression level pattern in plants grown under the two light types analyzed (Figure 4—*HFR1*). In particular, under the HPS light type, the gene expression level remained stable until 6 HAD and then subsequently decreased, reaching the minimum values (0.2-fold) at 24 HAD (Supplementary Table S4). Under the CoeLux light type, the *HFR1* gene showed an initial over-expression at 6 HAD, up to 2.8-fold the reference value, and a subsequent reduction to 0.2-fold at 12 and 24 HAD. A statistically significantly higher value was observed under the CoeLux^®^ light type, with respect to the HPS light type, at 6 HAD.

### 3.3. Loss-of-Function Mutant Plants

**Figure 5:**
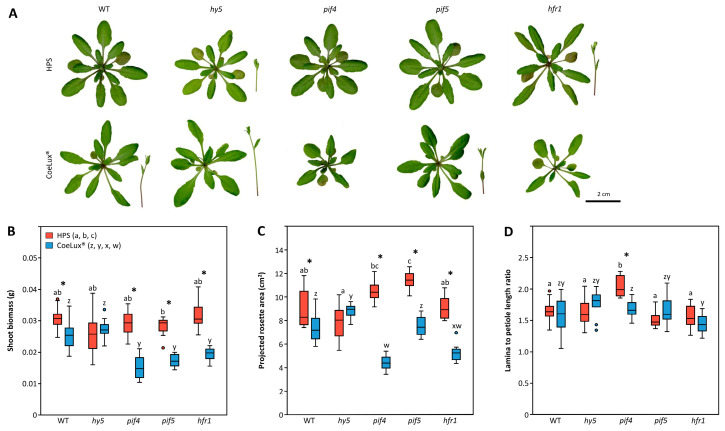
(**A**) Comparison of representative rosette phenotypes of wild-type (WT) plants and loss-of-function mutant lines for the *HY5*, *PIF4*, *PIF5*, and *HFR1* genes grown under both light types. (**B**) Shoot biomass (g), (**C**) projected rosette area (cm^2^), and (**D**) lamina-to-petiole-length ratio of mutants grown under both light types, in red under the HPS light type (control) and blue under the CoeLux^®^ light type. Boxes represent approx. 50% of the observations (*n* = 12 biological replicates), while lines extending from each box represent the upper and lower 25% of the distribution. Within each box, the solid horizontal line represents the median value, while circles denote outliers, colored red for the HPS treatment and blue for the CoeLux® treatment. Black asterisks denote statistically significant differences (*p* < 0.05) between plants grown under the CoeLux^®^ or the HPS light type, while letters denote differences between different mutants grown under the same light type.

The morphological analysis performed on loss-of-function mutant plants and their respective wild-type (WT) controls showed a general decrease in plant performance under the CoeLux^®^ light type (Figure 5A and Supplementary Table S5). In particular, WT plants growing under the CoeLux^®^ light type showed lower shoot biomass and projected rosette area (PRA) with respect to plants growing under the HPS light type (Figure 5B,C). At the same time, no differences were observed in the lamina-to-petiole-length ratio (L/P) (Figure 5D). Under the HPS light type, loss-of-function mutants showed no differences from the WT control, with the only exceptions being the *PIF5* gene mutant showing an increased PRA (Figure 5C) and the *PIF4* gene mutant showing an increased L/P (Figure 5D). On the contrary, under the CoeLux^®^ light type, loss-of-function mutants showed a general decrease in both shoot biomass and PRA compared to WT plants. The only exceptions are the *HY5* gene mutant, which showed no differences in shoot biomass and an increased PRA (Figure 5B,C), and the PIF5 mutant, which showed no differences in PRA (Figure 5C). Regarding the L/P of mutants grown under the CoeLux^®^ light type, all mutants showed no differences from the WT (Figure 5D).

When compared to the WT plants grown under the HPS light type, the mutants grown under the CoeLux^®^ light type showed a marked decrease in plant performance, with the only exception being the *HY5* gene mutant, which showed no differences in shoot biomass (*p* = 0.279), PRA (*p* = 1.000), and L/P (*p* = 0.994) (Figure 5B–D). In addition, the *PIF4* (*p* = 1.000) and *PIF5* (*p* = 1.000) mutants showed no differences in the L/P when compared to the HPS WT control (Figure 5D).

## 4. Discussion

By detecting light quality, intensity, and direction, photoreceptors and a multitude of downstream transcription factors play crucial roles in the plant’s adaptation to the unique light environment provided by the sun-like CoeLux^®^ lighting system.

The elongated Hypocotyl 5 (HY5) transcription factor plays a crucial role in regulating the development of plants in response to light, regulating both photomorphogenesis and shade-avoidance responses [9,10]. Previous studies demonstrated that, in darkness and low-light conditions, the level of HY5 is kept low due to degradation mediated by the constitutively photomorphogenic 1 protein (COP1), an E3 ubiquitin ligase that targets HY5 for proteasomal degradation [28]. In contrast, upon light exposure, photoreceptors suppress COP1 activity, allowing for higher levels of HY5 to accumulate in the nucleus and initiate the transcription of light-responsive genes, thus promoting photomorphogenic changes [9]. Although the regulation of light-inducible genes mediated by HY5 has been well investigated, the gene expression and regulation of *HY5* itself are still partly unexplored. A previous study showed that, in 3-day-old *A. thaliana* seedlings, the *HY5* gene expression level was low at the end of the night, while high levels were measured already 2 h after the beginning of the day, irrespective of shade or sun conditions [23]. Subsequently, under shade conditions, the levels of expression of the *HY5* gene decreased during the day, while exposure to a sun condition was able to re-establish high levels of gene expression [29]. These observations contrast with what we observed on 17-day-old *A. thaliana* leaves in the short-term light treatment (STLT). Indeed, we observed higher expression levels at the end of the night and a decrease in expression levels in the first 6 h after the lights were turned on. Afterward, we observed a slow expression level increase until the 24 h time point. Under the CoeLux^®^ light type, a significantly lower *HY5* expression level was measured at the 2 h time point, which could result in the greater suppression of photomorphogenesis compared to control plants and partially supports our first hypothesis. In control plants of the long-term light treatment (LTLT), we observed a higher *HY5* expression level in plants growing at the highest light intensities, supporting our first hypothesis. These observations are in accordance with what was previously observed at the protein level [9,30] and suggest that the plants growing at higher light intensities promote photomorphogenesis responses regulating both HY5 protein degradation and *HY5* gene expression level [30]. As hypothesized, under the CoeLux light type, a lower *HY5* expression level with respect to the control was observed both at 120 and 70 μmol m^−2^s^−1^, while, surprisingly, a higher expression level was observed at the lowest light intensity (i.e., 30 μmol m^−2^s^−1^). In previous studies, seedlings of the *hy5* mutant were reported to show partially etiolated phenotypes at various wavelengths of light [30]. However, we could not observe a similar phenotype in 25-day-old plants under both light types. At this developmental stage, no differences with respect to the wild type (WT) were observed in both shoot biomass and lamina-to-petiole-length ratio (L/P). In contrast, an increased projected rosette area (PRA) was observed in *hy5* plants growing under the CoeLux^®^ light type, revealing an opposite response to that which was expected. In this context, we considered the presence of the HY5 homolog (HYH) gene, encoding a bZIP transcription factor that shares significant sequence similarity with HY5 and can partially compensate for HY5 loss of function [31]. However, this compensation was described as context-dependent, as HYH’s activity is more pronounced under certain light conditions, such as high-intensity light or UV-B exposure [32,33], that do not align with the conditions set in this experiment’s treatments. Nevertheless, other transcription factors within the photomorphogenic network may exhibit functional redundancy with HY5, compensating for its reduced activity and thereby buffering the phenotypic impact. Furthermore, *hy5* mutants grown under the CoeLux^®^ light type were the only mutants that showed no differences in biomass and PRA compared to WT plants grown under the control light type, suggesting *HY5* as a suitable candidate gene for further studies aimed at explaining the mechanisms behind these observations and potentially improving plant growth under the CoeLux^®^ lighting systems.

Since HY5 is the central hub of the transcriptional network that regulates photomorphogenesis, a large number of proteins act to regulate HY5 function [10]. COP1 plays a crucial role, as many photoreceptors accomplish their signaling functions by regulating COP1 activities [34]. In particular, light conditions induce phytochromes (PHYA and PHYB) and cryptochromes (CRY1 and CRY2) to inhibit COP1 activity. Moreover, HY5 itself promotes the expression of its negative regulator, COP1 [35]. In this context, in the LTLT, we expected to observe higher *COP1* gene expression levels at lower light intensities; however, no significant gene expression-level variations were observed between light intensities or light type, rejecting our second hypothesis and suggesting that, in leaves of mature *A. thaliana* plants, the COP1 levels’ modulation takes place at the protein level. Conversely, in the STLT, we observed a significantly lower gene expression level beginning 6 h after dawn, consistent with the expected results. Furthermore, under the HPS light type, we observed a return to higher *COP1* gene expression levels at the end of the light treatment, in accordance with previous studies reporting an increase in *COP1* expression levels at the end of the day [36]. This mechanism allows COP1 to suppress transcription factors that promote photomorphogenesis, like HY5 and long hypocotyl in far-red 1 (HFR1), when entering dark conditions [36].

HFR1 is considered a positive regulator of photomorphogenesis [12] and a negative regulator of shade-avoidance responses via the inhibition of the phytochrome-interacting factors (PIFs) PIF4 and PIF5 [37]. When plants are exposed to low-light conditions with a high far-red light component (e.g., under a canopy or in dense plant stands), the *HFR1* gene expression level increases. This upregulation is known to be triggered by the activation of phytochrome A (PHYA), which senses the red-to-far-red-light ratio (R/FR) [38] and cryptochrome 1 (CRY1), which senses blue light attenuation caused by competition with other plants [39]. Together, these photoreceptors regulate shade-avoidance responses, inducing *HFR1* gene expression level; limiting HFR1 protein degradation through COP1 [40]; and promoting a balanced growth response by preventing excessive stem elongation, which can be detrimental if low light levels persist [41]. Despite the low number of biological replicates, the results of the LTLT clearly confirm our third hypothesis and show that the expression level of the *HFR1* gene is maintained at very high levels at lower light intensities, demonstrating that light intensity alone is sufficient to activate the expression of this gene to inhibit detrimental responses during long suboptimal light conditions. Furthermore, at the 30 and 70 μmol m^−2^s^−1^ light intensities, the *HFR1* gene showed a higher level under the CoeLux^®^ light type with respect to the HPS light type, supporting our third hypothesis. Since the CoeLux^®^ light type is not characterized by lower R/FR when compared to control [22], we speculated that this response could have been generated by the low blue light levels characterizing the CoeLux^®^ light type. Further confirmation was given by the STLT, where a higher *HFR1* expression level was observed under the CoeLux^®^ light type 6 h after dawn. With respect to the photoreceptor’s expression levels assessed in our previous study [18], we observed similar expression level trends between *HFR1* and the two photoreceptors that mainly regulate its expression and activity [40]. Indeed, in the LTLT under the HPS light type, both *PHYA* and *CRY1* showed an increased expression level at the lower light intensities, suggesting the existence of a direct relation. However, even if the gene expression of the *HFR1* gene is significantly induced by low light, it cannot be concluded that the HFR1 protein accumulates under low-light conditions. Further regulation will occur at the protein level, e.g., due to COP1-mediated degradation, thus reducing the level of functional protein and potentially leading to extremely low protein levels. The *hfr1* mutant was reported to exhibit an exaggerated elongation in low-light and shaded environments due to its impaired suppression of shade-avoidance responses [42,43]. However, no differences in L/P were found when *hfr1* mutant plants were compared to WT plants. Meanwhile, as expected, lower biomass and PRA were observed under the CoeLux^®^ light type, partially supporting our fourth hypothesis and in accordance with previous studies that reported smaller cotyledons and leaf area in *hfr1* mutants [41].

HFR1 counteracts the elongation-promoting effects of PIFs by forming non-DNA-binding heterodimers with PIF4 and PIF5, which are also upregulated in shade, and promote shade-avoidance responses by directly binding to the G-boxes present in the promoters of shade-induced genes [44]. At the transcriptional level, the PHYB, CRY1, and CRY2 photoreceptors were observed to directly interact with PIF4 to restrict its transcriptional activity [45,46]. As hypothesized in our second hypothesis, in the LTLT, the PIF4 gene showed a clear trend, resulting in a higher expression level at lower light intensities, confirming the involvement of this gene in responses to low light levels and regardless of R/FR. This trend reflects the observations previously made at the protein level, where PIF4 and PIF5 were reported to be abundant in dark and shade-mimicking conditions [47]. However, unlike our second hypothesis, no expression level differences were observed between plants grown under the two diverse light types. In contrast with *PIF4*, despite showing an expression level increase at 30 and 70 μmol m^−2^s^−1^ under the HPS light type, the *PIF5* gene has not demonstrated a similar trend, revealing a more complex regulation mechanism, probably at the protein level. The expression levels of the *PIF4* and *PIF5* genes are regulated by the circadian clock and were reported to peak at the end of the night [48]. Even though the *PIF4* and *PIF5* transcript levels may remain relatively high when light returns, the protein levels decrease rapidly due to phytochrome-mediated degradation in response to red light and high R/FR [47,48]. However, in the STLT, the expression levels of both genes showed a diverse trend, registering an increase in the first hours after exposure to light and reaching very high levels, as in the case of *PIF4* 6 h after dawn. These data support previous observations [48], highlighting that transcript levels are not the only factor determining growth in response to changing light conditions; indeed, regulation mediated by a wide variety of actors at the protein level plays a pivotal role. With respect to the photoreceptor’s expression levels assessed in our previous study [25], we observed similar expression level trends between the *PIF4* gene profile and two of the photoreceptors that were described to regulate its expression level and activity [45,46]. Indeed, in the LTLT under the HPS light type, both *CRY1* and *CRY2* showed an increased expression level at lower light intensities, similar to the expression level of the PIF4 gene, suggesting a direct relationship. *Pif4* mutants were reported to be hypersensitive to red light, failing to exhibit the full shade-avoidance syndrome in suboptimal light conditions [14,42]. Supporting these data, *pif4* mutants showed an increased L/P with respect to the WT under the HPS light type. However, the same increase was not observed under the CoeLux^®^ light type, while a marked reduction was observed in both biomass and PRA, consistent with our fourth hypothesis. Similarly, *pif5* mutants were also reported to be hypersensitive to red light, exhibiting shorter hypocotyls and larger cotyledons than WT plants, even in low-light conditions [49]. Supporting these data, we observed a higher PRA with respect to WT plants grown under the HPS light type in our 25-day-old *pif5* plants. However, under the CoeLux^®^ light type, no differences were observed in PRA and L/P, while lower shoot biomass was observed.

## 5. Conclusions

Our findings demonstrate that the CoeLux^®^ light type, with its distinct blue-to-green ratio and reduced blue light levels, stimulates responses in *Arabidopsis thaliana* plants comparable to those displayed in shade conditions. The regulation of these responses involves key photoreceptors and transcription factors, particularly HY5, COP1, HFR1, and PIFs, which collectively orchestrate light-responsive growth patterns. Our results demonstrate that both transcriptional and protein levels combine to tune the plant’s responses to suboptimal light conditions. In detail, we found that the *HY5* gene expression level patterns differ significantly under the CoeLux^®^ light type compared to HPS lighting, especially in response to light intensity, suggesting that the CoeLux^®^ light type suppresses photomorphogenic growth via altered HY5 activity. On the contrary, COP1’s gene expression level changes were not observed under varying light conditions, at least in mature *A. thaliana* leaves. Notably, the *HFR1* gene exhibited a higher expression level at low light intensities and under the CoeLux^®^ light type, consistent with its inhibitory role in shade-avoidance responses. In contrast, the influence of the CoeLux^®^ light type on PIFs expression levels seems more marginal. Future studies with additional replicates will help strengthen these conclusions. Nevertheless, these data emphasize the complex interplay between light quality and intensity, photoreceptor signaling, and transcriptional responses in shaping plant morphology and development. Furthermore, these results identify the *HY5* and *HFR1* genes as potential targets for optimizing plant growth in controlled environments enlightened with CoeLux^®^ or other advanced lighting systems, highlighting the need to explore further transcriptional and protein-level responses with the aim of refining plant adaptation to artificial light environments.

## Figures and Tables

**Table 1:** Spectra color composition of the CoeLux^®^ and HPS light types. The sum of normalized photon counts was calculated for each color interval and reported in the form of relative intensity ± SD (*n* = 8).

Color	Wavelength Range (nm)	Relative Intensity (%)	
		CoeLux^®^	HPS
Blue	400–490	14.02 ± 0.57	23.62 ± 0.36
Green	490–560	24.12 ± 0.17	24.22 ± 0.64
Yellow	560–590	14.47 ± 0.47	10.94 ± 0.12
Red	590–700	41.06 ± 0.51	34.71 ± 0.56
Far-red	700–780	6.33 ± 0.19	6.50 ± 0.12

**Table 2:** List of primers used in this study.

Gene	Locus	Primer Sequence (5′ > 3′)	Source
*HFR1*	AT1G02340	AGTGATGATGAATCGGAGGAGTT	This study
		CCGAAACCTTGTCCGTCTTG	
*COP1*	AT2G32950	GGGAAGCACTACAAAGGGGT	This study
		CTGGAGATCAGTTTGCACCTCA	
*HY5*	AT5G11260	AAGCGGCTGAAGAGGTTGTT	This study
		TCCAACTCGCTCAAGTAAGCC	
*PIF4*	AT2G43010	AACGGACTCATGGACTTGCT	This study
		TGGTGTTCCATGTCAGATCTAAGG	
*PIF5*	AT3G59060	AATCTTCCATCCATTCAGAGGCT	This study
		TCCACTAATTCATCTTCTGGTCTGA	

## Data Availability

Data are contained within the article or Supplementary Materials. Further inquiries can be directed toward the corresponding author.

## Supplementary Materials

The following supporting information can be downloaded at https://www.mdpi.com/article/10.3390/biology14101315/s1, Supplementary Table S1: stability of reference genes (PP2AA3, UBQ10, and SAND) across all treatments of the LTLT; Supplementary Table S2: stability of reference genes (PP2AA3, UBQ10, and SAND) across all treatments of the STLT; Supplementary Table S3: Data regarding the long-term light treatment (LTLT) presented in Figure 3; Supplementary Table S4: Data regarding the short-term light treatment (STLT) presented in Figure 4; Supplementary Table S5: Data regarding the loss-of-function mutant lines phenotype presented in Figure 5.
