# Supplementary material for: The Gene Expression of the Transcription Factors HY5 and HFR1 Is Involved in the Response of Arabidopsis thaliana to Artificial Sun-like Lighting Systems
Source: Biology (Basel). 2025 Sep 23;14(10):1315. doi: 10.3390/biology14101315 (PMC12561826; doi:10.3390/biology14101315)
Supplement: Supplementary file 1 [file biology-14-01315-s001.zip › biology-3824557-supplementary.pdf]

Supplementary Table S1: stability of reference genes (PP2AA3, UBQ10, and SAND) across all treatments of the LTLT. For each gene, mean Ct, standard deviation (SD), minimum (Min) and maximum (Max) Ct values, and coefficient of variation (CV%) are reported.

**Gene**	**Mean Ct**	**SD (Ct)**	**Max Ct**	**Min Ct**	**CV (%)**
PP2AA3 (AT1G13320)	26.32	0.37	27.14	25.80	1.41
UBQ10 (AT4G05320)	20.94	0.29	21.41	20.30	1.39
SAND (AT2G28390)	27.53	0.25	28.21	27.25	0.91

Supplementary Table S2: stability of reference genes (PP2AA3, UBQ10, and SAND) across all treatments of the STLT. For each gene, mean Ct, standard deviation (SD), minimum (Min) and maximum (Max) Ct values, and coefficient of variation (CV%) are reported.

**Gene**	**Mean Ct**	**SD (Ct)**	**Max Ct**	**Min Ct**	**CV (%)**
PP2AA3 (AT1G13320)	26.56	0.53	27.54	25.37	2.03
UBQ10 (AT4G05320)	21.26	0.32	21.98	20.57	1.50
SAND (AT2G28390)	28.03	0.45	29.00	27.18	1.63

Supplementary Table S3: Data regarding the long-term light treatment (LTLT) presented in Figure 3. The relative gene expression is reported as the mean ± standard deviation (SD).

**Treatment**	**HFR1**	**HY5**	**COP1**	**PIF4**	**PIF5**
HPS120	1,04 ± *0,33*	1,00 ± *0,04*	1,00 ± *0,08*	1,04 ± *0,34*	1,00 ± *0,07*
HPS70	3,34 ± *0,67*	0,83 ± *0,16*	1,53 ± *0,56*	4,19 ± *1,01*	3,07 ± *0,37*
HPS30	31,04 ± *7,16*	0,15 ± *0,01*	0,94 ± *0,32*	6,85 ± *2,15*	1,70 ± *0,46*
CoeLux120	1,43 ± *0,75*	0,44 ± *0,10*	1,73 ± *1,32*	3,02 ± *2,35*	3,46 ± *1,81*
CoeLux70	14,56 ± *3,46*	0,36 ± *0,04*	1,25 ± *0,34*	4,42 ± *2,45*	2,19 ± *0,19*
CoeLux30	250,36 ± *75,92*	0,44 ± *0,07*	0,95 ± *0,33*	5,92 ± *2,15*	1,98 ± *0,17*

Supplementary Table S4: Data regarding the short-term light treatment (STLT) presented in Figure 4. The relative gene expression is reported as the mean ± standard deviation (SD).

**Treatment**	**HFR1**	**HY5**	**COP1**	**PIF4**	**PIF5**
t0 HPS	1,03 ± *0,27*	1,00 ± *0,09*	1,00 ± *0,07*	1,15 ± *0,67*	1,01 ± *0,18*
t2 Coelux	1,26 ± *0,43*	0,41 ± *0,02*	0,94 ± *0,14*	57,04 ± *41,34*	2,33 ± *0,26*
t2 HPS	0,79 ± *0,31*	0,52 ± *0,05*	1,25 ± *0,16*	42,86 ± *25,29*	2,71 ± *0,39*
t6 Coelux	2,79 ± *1,01*	0,14 ± *0,01*	0,62 ± *0,14*	70,67 ± *43,55*	1,62 ± *0,73*
t6 HPS	1,02 ± *0,17*	0,13 ± *0,02*	0,48 ± *0,11*	55,59 ± *28,59*	1,19 ± *0,39*
t12 Coelux	0,19 ± *0,17*	0,28 ± *0,03*	0,63 ± *0,22*	21,77 ± *14,52*	0,22 ± *0,10*
t12 HPS	0,32 ± *0,15*	0,17 ± *0,01*	0,63 ± *0,27*	20,28 ± *7,92*	0,22 ± *0,10*
t24 Coelux	0,17 ± *0,04*	0,46 ± *0,08*	0,59 ± *0,17*	2,28 ± *1,93*	0,25 ± *0,14*
t24 HPS	0,18 ± *0,12*	0,43 ± *0,04*	0,98 ± *0,28*	8,15 ± *2,51*	0,86 ± *0,38*

Supplementary Table S5: Data regarding the loss-of-function mutant lines phenotype presented in Figure 5. The data are reported as the mean ± standard deviation (SD).

Treatment and mutant	Shoot biomass (g)	PRA (cm^2)	L/P
HPS_WT	0,014 ± 0,003	4,951 ± 0,546	1,407 ± 0,273
HPS_HFR1	0,015 ± 0,003	5,190 ± 0,933	1,458 ± 0,127
HPS_HY5	0,024 ± 0,005	8,737 ± 1,090	1,529 ± 0,107
HPS_PIF4	0,017 ± 0,003	6,564 ± 0,604	1,746 ± 0,131
HPS_PIF5	0,020 ± 0,004	7,448 ± 0,793	1,547 ± 0,127
CoeLux_WT	0,016 ± 0,002	4,316 ± 0,732	1,488 ± 0,216
CoeLux_HFR1	0,017 ± 0,004	4,691 ± 0,974	1,428 ± 0,203
CoeLux_HY5	0,016 ± 0,003	3,319 ± 0,457	1,491 ± 0,183
CoeLux_PIF4	0,011 ± 0,001	2,671 ± 0,268	1,954 ± 0,233
CoeLux_PIF5	0,012 ± 0,002	2,722 ± 0,451	1,633 ± 0,302
